# Psychology of Acute Rheumatism

**Published:** 1857-11

**Authors:** Albert T. Wheelock

**Affiliations:** Author of Boylston Prize Essay for 1838, &c. &c.; Belfast, Maine


ART. III.—*Psychology of Acute Rheumatism.* By ALBERT T. WHEELOCK, A.M., M.D., Author of Boylston Prize Essay for 1838, &c. &c. Belfast, Maine.

THIS title is selected, because it is as convenient as any other, for the purpose of an *exposé* of the *mental and moral conditions* connected with the access of acute rheumatism.

American physicians, as a class, have given comparatively much attention to the means of preventing disease, and to hygiene, or the moral, mental, and physical methods of cure apart from the use of medicine. Eminently an inventive people, may we not justly expect very important improvements here, in consequence of this especial characteristic. The result may be to lessen the number of physicians needed by the community. There may be less medicine purchased and used. Truth, nevertheless, in this country of all countries in the world, is never to be feared. The physiological knowledge, now in wide practical diffusion, is mainly of course due to the researches of scientific medical inquiry. The race is already improved, and is being more so, at the present time, from the same cause; how far these inquiries are to extend and the consequent improvements to continue, we can judge only from analogies. What more noble and philanthropic, then, than to lead one's exertions to still farther multiply this kind of truth, resting quite assured that what will benefit the masses will enure to universal mutual benefit.

To prevent disease, it may, of course, be generally necessary that the cause be understood. Not that many of the causes of contagious diseases may be removed in the present state of society,—such, for example, as rubeola, parotitis, scarlatina,—the direct cause being a proximity to the affected; the result, a disease of the like kind with that of the previously afflicted person.

From an improved medical science, the causes of all diseases *may* be as well understood as is now the producing cause in well-known contagious maladies; that is, the *rationale* of the production of any given disease may be discovered, simply and thoroughly explained, and demonstrated.

For the last hundred years no definite improvement has been made in this regard. Indeed, is the cause of any of the inflammatory diseases settled upon sufficient and thorough observation, so that no farther proof is called for to satisfy a philosophical observer? Is there not a multitude of causes laid down with more or less indefiniteness, to which is ascribed the production of any one of these diseases? Is not our information unsatisfactory, when compared to a definite, mathematical, and invariable statement of the cause?

A definite cause discovered directly producing any one of these diseases, affords a valuable stand-point from which to make similar discoveries. At least it affords encouragement that may lead to like observations in regard to some other diseases.

Needful observations, careful, definite, and intelligent, from the peculiar nature of such inquiries, are not easily made, so many circumstances, such as varieties of climate, varieties of habits, varieties of constitution, hereditary or acquired, are to come into account. There is this consolation, however, for those who would make such observations, that there are at this time a large number, and that number constantly increasing, who will understand and appreciate; and that superficial inquiries, based on ill-considered or too small a number of facts, or, in reality, on none at all but visionary ones, do not long hold competition with them; that the time is gone by when mere theories plausibly maintained are to be received as of equal value with opinions based upon facts and laborious investigations.

The materials in reference to the disease named at the beginning of this article, collected during a period of twenty years of professional experience, consist of observations of fifty-four cases of acute rheumatism. The patients were sufficiently intelligent, and the inquiries, new to them as well as to the other attendants, were, no doubt, altogether unexpected, but at once understood, and their propriety and pertinency admitted; all these inquiries and statements became confirmatory of a principal truth, viz.:

EVERY ACCESS OF ACUTE RHEUMATISM IS IMMEDIATELY PRECEDED BY A SPECIAL MENTAL CONDITION.

Nothing of the kind has been, to my knowledge, laid down by any of the medical writers of England or America; and during the past season an opportunity was afforded to make personal inquiries of several leading physicians in Paris, who, with unanimity, confirmed my previous belief, that nothing like it had been recorded by the best-known writers of France or Germany. The medical opinion, based on the accompanying facts, was then written by me in the French language, and published under the auspices of the Minister of Public Instruction.

The theory is, that cold and mental agitation PRODUCE acute rheumatism.

The cases given, selected not entirely at random, but to show the production of the disease in various aspects, were well marked, attended by fever at the outset, followed by extreme muscular prostration, and continued generally several months.

Obs. I.—G. P., mechanic, age 19, an orphan, had, by several years of industry, saved a few hundred dollars, which he had lent to a friend in mercantile business. On learning that he was likely to lose his hardly-acquired fortune, which was his all, as far as money was concerned, was very anxious respecting it, both by fear of losing it and in devising means in regard to it, so much so as to have remained without sleep or appetite for several days successively. During the period passed in this manner, by some neglect, he became exposed to a sudden suppression of the perspiration by cold. A severe attack of rheumatism immediately came on, attended by violent pain in several parts of the body, and by extreme muscular prostration; disease continuing several months.

Obs. II. W. A. O., aged 40, unmarried, while on a voyage from Liverpool to Boston, the latter part of the winter season, was in a gale of wind for fifteen days, and during the whole of that time constantly apprehending serious injury to the ship, or a complete and fatal shipwreck. He had made his own fortune, had always been heretofore successful, and was a large owner in the ship. During the first days of this stormy period he was much exposed to the weather, and of course very anxious concerning the present situation of himself and crew. At this time he experienced a severe cold, and a few days after was prostrated by an attack of acute rheumatism. On arrival in port, being removed to the hospital, he remained some months there, and, on discharge, continued incapable for business several months thereafter: finally, complete recovery.

Obs. III. C. A., aged 25, had an intimacy with a near relative, and she had become pregnant by him. Both were well known to a large circle of acquaintance, and in a situation rendering the consequences likely to be publicly exposed. Planning various methods of concealment, and attempting to carry them into execution, under the greatest mental excitement and anxiety, he became exposed to severe cold, rapidly followed by an attack of acute rheumatism, the more serious effects of which lasted several months.

Obs. IV. E. T., nurse, aged 30, unmarried, of much valuable experience in her present vocation, was to attend her sister during an approaching confinement, generally unusually critical. Learning by telegraph that her sister had been confined prematurely, she was exceedingly anxious to obtain an immediate conveyance to her. While under this mental excitement, making unaccustomed exertions to succeed in her object, neglecting usual precautions, she experienced a sudden suppression of the perspiration by cold, and suffered a most severe attack of rheumatism; was in the greatest agony of pain for several days, soon followed by extreme prostration.

Obs. V. B. A., clergyman, aged 40, married, nervous sanguine temperament, bilious habit, of unusually social disposition, happy in his domestic relations, accomplished in education, lost, by sudden death, an only child, a son of eight years. The affliction produced nervous excitement amounting to partial insanity. While attending to some customary manual labor and exercise, during this mental disturbance, he became wet in a shower of rain, and, neglecting precautions, experienced a severe cold. Rheumatism succeeded, attended with extreme mental and bodily prostration for some months.

Obs. VI. J. K., farmer, aged 20, married, living in a newly-settled district, clearing up his land in the forest and making a farm, was laboring every day in the week to have his crops sown in suitable season. Having conscientious scruples against working on the Sabbath, he suffered much anxiety and self-reproach on that account. During this time, a severe cold, in consequence of the negligence produced by his distracted state of thoughts, preceded an attack of rheumatism of five months' duration.

Obs. VII. M. C., merchant, aged 35, married, contracted gonorrhœa from a woman of the town. While suffering anxiety of mind, in much fear of the discovery of his faithlessness by his wife and friends, and attending to customary employment, by some neglect he experienced a severe cold, which was immediately succeeded by an access of acute rheumatism of several months' continuance.

Obs. VIII. G. B. M., medical student, aged 23, was candidate for house-physician to a hospital, which position was to be the consequence of successful oral examination and thesis. Being embarrassed pecuniarily, he much desired the situation, and also because he considered it honorable under the circumstances. He was for six months engaged in preparing the dissertation. As the examination approached, he exerted himself very laboriously in his studies, and had much anxiety in regard to the result. Succeeding some active physical exertions, he suffered a sudden diminution of the vital heat of the body by cold, which was immediately followed by a lengthened attack of acute rheumatism, several partial returns of which were subsequently experienced.

Obs. IX. C. H., a jeweller, aged 26, had pursued his occupation successfully for two years in a new and flourishing city, when, being obliged to vacate his premises in consequence of the sale of the building, he found much difficulty in obtaining another. He spent several days unsuccessfully in inquiring about the place for that object. He was of a somewhat delicate habit physically, made more so by sedentary employment within doors; and while exerting himself in this unusual manner, being much depressed and anxious at the time, he became exposed to sudden cold, the consequence being an access of acute rheumatism, which recurred at several subsequent periods of his life.

Obs. X. J. R., female, married, aged 29, of an amiable and affectionate disposition, temperament nervous, of unusual sensibility, had been for two or three weeks making preparations for an entertainment of numerous friends at her house, in a place where little assistance of the kind required could be obtained conveniently. As might be expected in a person of such characteristics, she was quite anxious that everything should pass off to entire satisfaction, and, during the evening of the entertainment, was exposed to a strong draft of air while receiving her guests as they successively arrived through the opened doors. During the evening she was able to attend to the duties as hostess; the following day commenced an attack of rheumatism, the disease continuing through the period of four months.

Obs. XI. B. W., a young professional man, aged 24, had an urgent engagement several miles from his residence, and was unjustly disappointed in his expected means of conveyance thither. While very much vexed, and anxious about the consequences of his delay, he went hurriedly to seek another conveyance about the town, and obtaining a private carriage, he jumped into it in a state of high perspiration and drove rapidly off. The next day was the commencement of a severe and lengthened rheumatic attack, the case being attended with the usual pain, and physical and mental prostration.

Observations similar to the preceding, five times in number, made in a series of years, affording no exception against the proposition above enunciated, its converse also being equally found true, that no case of rheumatism has been observed not traceable to a special mental disturbance previous, have settled indubitably, in my opinion, the connection between the phenomena described and the disease, acute rheumatism.

What, then, is one of the most important indications in treatment? It is of course to bring into operation the requisite moral influences. The patient is to be made to understand the true nature of the disease and its cause; a reaction is very frequently produced thus, at the discovery and belief that the disease has been partly or entirely brought on by indulgence in mental anxiety, which, to say the least, may not be necessary, and may and frequently should be avoided. It cannot be expected that every individual shall exercise the philosophical calmness requisite to forgetting or ignoring mental agitations; but from the nature of the case, it may be presumable that a knowledge of the really producing cause of the disease will greatly assist in fortifying the sufferer against a protracted continuance of it. In my own experience of its treatment, I have found that when patients are informed that it has been brought on themselves by a mental agitation, that might seem to be avoidable or inexcusable and needless, the disease has been shortened in its course or immediately stopped; and in cases where there have been successive attacks, on the patient being informed of their nature and cause, he has apparently been thus spared these recurring attacks.

The preceding inquiries are no doubt in the true spirit of the medical profession, which ranks the means of preventing disease, or lessening its violence among its most important discoveries. If there is less confidence in specifics; if there is more known of the deleterious effects of some kinds of medication; if the number of diseases found to be uncontrolled by medicine, is an increasing catalogue, it is owing to conscientious and carefully noted observations by the faculty. Vaccination, the forestalling of dyspepsia, the principles and necessity of ventilation, may be proudly signalized as benefits which, however properly appreciated, have never been recompensed by the vulgar, who suppose all motives to end in the expectation of pecuniary reward. This kind of information by the press, and by individual advice to patients, is being more and more diffused, till all these subjects, so intimately connected with happiness, are more understood; and the people, by avoiding many errors already known, are learning that the good, the true, and the beautiful, are one and the same: that perfection of form even is to follow mental and moral perfection, till a new trinity of beauty, love, and worship is to be added to the sublime dogmas of received revelation, in the new Evangel of health.

